# Plasma Vascular Endothelial Growth Factor Dysregulation in Defining Aggressiveness of Head and Neck Squamous Cell Carcinoma

**DOI:** 10.1155/2012/687934

**Published:** 2011-12-15

**Authors:** Boonchu Kulapaditharom, Vipa Boonkitticharoen, Chanika Sritara

**Affiliations:** ^1^Department of Otolaryngology, Ramathibodi Hospital, Mahidol University, 270 Rama VI Road, Bangkok 10400, Thailand; ^2^Department of Radiology, Ramathibodi Hospital, Mahidol University, 270 Rama VI Road, Bangkok 10400, Thailand

## Abstract

*Background*. High circulating vascular endothelial growth factor (VEGF) levels tend to reflect tumor aggressiveness for being associated with tumor progression and prognosis. Measurement of soluble VEGF receptor-1 (sVEGFR-1) may improve diagnostic power of VEGF assay. *Methods*. This study investigated regulation of plasma VEGF by sVEGFR-1 in 82 patients with head and neck squamous cell carcinoma compared with 32 healthy subjects to obtain information for assay characterization. *Results*. Normality or abnormality of VEGF/sVEGFR-1secretion patterns was rated into five diagnostic levels from definitely abnormal (likelihood ratios) (LRs = 4–∞) to definitely normal (LRs = 0–0.17). Because of ineffective VEGF regulation, high grade tumor had a greater chance (62.5%) than low grade tumor (20%) in expressing a definitely abnormal pattern and a lower chance to express the normal pattern (*P* = 0.007). VEGF alone had much lower diagnostic power in differentiating between normal (LRs = 0.3–0.9) and abnormal secretion patterns (LRs = 2.2–2.4). *Conclusions*. VEGF dysregulation is suggestive of tumor aggressiveness for causing persistent plasma VEGF elevation. sVEGFR-1 improves diagnostic power of VEGF assay particularly in identifying subset of low grade tumor with underlying aggressive disease and ruling out aggressiveness in subset of high grade tumor.

## 1. Introduction

Head and neck squamous cell carcinoma (HNSCC) represents an aggressive epithelial malignancy. Most patients, 60−80%, are present with locally advanced disease at diagnosis [1]. Distant metastasis at the time of presentation is less common [2]. Metastasis to neck nodes is the most important prognostic factor, and advanced clinical stage is frequently linked to poor survival [3]. The tumor-node-metastasis (TNM) staging provides an anatomical roadmap for tumor site, size, and lymphatic involvement. Prognostic indicators derived from the TNM system are usually insufficient in explaining the disease aggressiveness for HNSCC. Patients with positive nodes may show different survival profiles or patients with negative nodes may be at risk of local relapse [4]. Biomarkers capable of identifying patients with biologically aggressive neoplasm would provide much-needed information for improving treatment accuracy and efficacy.

Angiogenesis is crucial for tumor growth, invasion, and metastasis [5]. Vascular endothelial growth factor (VEGF), a major regulator of angiogenesis and vascular permeability, plays a key role in pathogenesis of HNSCC [6, 7]. Up to 90% of HNSCCs express VEGF and its receptors, VEGFR-1–3 [6, 7]. Multiple studies on HNSCC demonstrated the association of VEGF with tumor progression [8, 9], lymph node metastasis [10, 11], and patient survival [6]. VEGF is not only an independent prognostic indicator for HNSCC [6] but also a potential target for anti-VEGF therapy [7]. Generally, tissue VEGF expression is a measure for tumor angiogenic activity but tissue measurement has certain disadvantage for being invasive and not lending itself for dynamic assessment of the angiogenic process. Detection of VEGF in blood circulation of cancer patient offers an alternative means for measurement. Blood sampling is noninvasive and can be performed at repeated intervals. In fact, shedding of tumor-derived VEGF into systemic blood serves to recruit bone marrow myeloid cells to provide additional angiogenic factors to the tumor organ for growth and progression [12]. Extensive studies in several tumor types including head and neck cancer [13–16], nonsmall cell lung cancer [17, 18], liver cancer [19], renal cancer [17], colorectal cancer [17], and ovarian cancer [20] revealed the correlation of elevated circulating VEGF with tumor tissue expression [14, 17], increased microvessel density [14], disease aggressiveness including lymph node metastasis, advanced clinical stage [14, 15], distant metastasis [16], and poor prognosis [13, 15, 17–20]. Despite these promising findings, circulating VEGF assay is less well characterized for its sensitivity and specificity in identifying patient with aggressive tumor. High false-positive and negative findings are documented in many reports [16, 21, 22]. An understanding of VEGF regulation in the systemic blood may help assay optimization and data interpretation.

 VEGF represents the angiogenic switch which may confer a metastatic potential to tumor even at the early stage of growth. Further tumor progression is controlled by the balance between positive and negative regulators of angiogenesis [5]. Availability of active VEGF is regulated by its soluble receptor-1 (sVEGFR-1) via ligand trapping [23]. In physiologic context (e.g., exercise), sVEGFR-1 is upregulated in responding to the rise in VEGF during exercise and is responsible for normalizing the VEGF level to baseline [24]. In pathophysiologic context (e.g., cancer), sVEGFR-1 is frequently coexpressed with VEGF [25–27]. Experimental and clinical data are suggestive of the possible role of sVEGFR-1 in inhibiting VEGF-induced tumor angiogenesis and tumor growth [25–28]. sVEGFR-1 level in excess of VEGF level is an indicator of good prognosis [25–27]. On this basis, we hypothesized that the relative VEGF and sVEGFR-1 levels might be an important factor in determining the progression of tumor from a less aggressive to a more aggressive stage. In this study, we conducted a cross-sectional study of cases and controls to analyze plasma VEGF/sVEGFR-1 secretion profiles to understand how plasma VEGF was regulated in patients with HNSCC compared with normal controls. The study variables included TNM stage, T category, nodal status, and tumor grade.

## 2. Materials and Methods

### 2.1. Study Population

The study population consisted of 82 patients with HNSCC and 32 healthy controls. Newly diagnosed patients at the Department of Otolaryngology, Ramathibodi Hospital were consecutively recruited during June 2006 to May 2009. Patients with distant metastases, lymph node metastases from unknown primaries were excluded. Tumor staging was classified according to the TNM system established by the American Joint Committee on Cancer. The healthy volunteers, who had no history of a known neoplasm and no clinical evidence of vascular, metabolic and inflammatory diseases, were recruited among hospital staff and graduate students. Demographic data of the study population are presented in Table 1. The study protocol was approved by Ramathibodi Hospital Ethics Committee. Patients and healthy subjects signed the informed consents to participate in the study.

### 2.2. Plasma Collection and Assay

Six mL of peripheral blood was collected into a tube with EDTA anticoagulant and placed in an iced bucket. The blood samples from patients were taken before treatment. Plasma was prepared within 1 hour of blood collection by centrifugation at 4°C at the speed of 1000 g for 15 minutes. The plasma sample was aliquoted and stored at −25°C until assay.

 Concentrations of VEGF and sVEGFR-1 were measured using two commercially available ELISA kits designed to measure VEGF_121_, VEGF_165_, and sVEGFR-1 (Quantikine, R&D Systems, Abingdon, UK). The VEGF assay kit (DVE00) was reported to measure only unbound VEGF while the sVEGFR-1 assay (DVR100) was reported to measure total sVEGFR-1 [24, 29]. According to the manufacturer, the minimum detectable concentrations for VEGF and sVEGFR-1 were <9 and 5.01 pg/mL, respectively.

### 2.3. Statistical Analysis

Comparisons of continuous data were performed by the Mann-Whitney test and of categorical data by *χ* ^2^ test or Fisher exact test. All statistical tests were 2-tailed, and *P* < 0.05 was considered statistically significant. 

## 3. Results

### 3.1. Study Population Characteristics

This study enrolled patients with SCC in the nasopharynx (20.7%), oral cavity (28%), oropharynx (14.6%), hypopharynx (18.3%), and larynx (18.3%). Regarding TNM stage, 64 out of 82 patients (78%) had stage III and IV diseases. The averaged age of patients (58.4 years) was higher than controls (31.7 years) (Table 1). The median VEGF and sVEGFR-1 levels were also higher (*P* ≤ 0.03) (Table 2). The recruitment of younger and more female healthy subjects as controls (M/F = 7/25) was a point of concern. Regarding previous publications, no significant association of VEGF with age and sex was reported in many studies [14, 20, 22]. In addition, equilibrium distribution of VEGF/sVEGFR-1(VR) secretion profiles observed for this control group was similar to that of another control study by a group from our institute (*n* = 99, age (SD) = 49.3 (0.4) years, M/F = 60/39, personal communication).

### 3.2. VEGF/sVEGFR-1 (VR) Secretion Profiles

Physiologic angiogenesis is a tightly regulated process to maintain the dynamic equilibrium between proangiogenic and antiangiogenic factors [23]. Malignant tumor utilizes the same angiogenic process for progression by upregulating the proangiogenic factors and downregulating the angiogenic inhibitors [5, 23]. Therefore, median concentrations of VEGF (V) and sVEGFR-1(R) of the control group were used as cut-off for high (H) and low (L) secretion levels. Four patterns of VR secretion profiles were defined as V_H_R_L_, V_H_R_H_, V_L_R_H_, and V_L_R_L_ to project the rise of VEGF level (V_H_R_L_) followed by the upregulation of sVEGFR-1 (V_H_R_H_), then the suppression of VEGF by sVEGFR-1 (V_L_R_H_) and eventually both factors returning to baseline (V_L_R_L_). A balanced distribution of VR secretion profiles was observed in controls (Table 2). Subjects with elevated VEGF (28%), that is V_H_R_L_ profile, were in balance with subjects (25%) with a V_H_R_H_ profile. Normalization of VEGF level was evidenced by the shift of V_H_R_H_ (25%) to V_L_R_H_ (31%) and eventually to V_L_R_L_ (16%). This was in contrast to the observation in patients from which the distribution was skewed toward V_H_R_H_ profile (54.9%).

### 3.3. VEGF Regulation Regarding Tumor Stage and Grade

It has been demonstrated by cell culture study that tumor-derived VEGF is able to induce the expression of sVEGFR-1 [30]. Upregulation of sVEGFR-1 by VEGF represents a negative feedback mechanism in regulating the level of VEGF as shown by the exercise study [24]. In this study, sVEGFR-1 upregulation was demonstrated by the shift of V_H_R_L_ to V_H_R_H_ profile and VEGF suppression by the shift of V_H_R_H_ to V_L_R_H_ profile. In relation to controls, marked shifts from V_H_R_L_ to V_H_R_H_ were recognized in patients with less aggressive disease stages including stage I, II; T_1,2_; N− and low-grade tumor (G_1,2_) but the magnitudes of shifts were not large enough to reach significant levels (*P* ≥ 0.11) (Table 3). However, the regulative capability of sVEGFR-1in normalizing VEGF levels in these patients was similar to that of healthy controls (*P* ≥ 0.38) (Table 3). In contrast, significant sVEGFR-1 upregulation could be confirmed in patients with more aggressive disease stages (*P* ≤ 0.009), but the upregulated sVEGFR-1 failed to suppress VEGF levels in these patients with stage III, IV; T_3,4_; N+ and high grade tumor (G_3,4_) (*P* ≤ 0.02).

 Since TNM variables and tumor grade were related to VEGF regulation it might be more relevant to analyze the effect of VEGF regulation in association with stepwise increase in combined tumor grade and stage to obtain an alternative point of view of VEGF regulation during the transition of tumor from low grade at an early state to high grade at the advanced stage. In this part, VR secretion profiles along with absolute concentrations of VEGF and sVEGFR-1 were determined for healthy controls, patients subgroups categorized according to the above elaboration (Table 4). Interestingly, median sVEGFR-1 levels in patients were significantly higher than that of controls for all steps of disease transition (*P* ≤ 0.04). But the elevated sVEGFR-1 could normalize the VEGF levels only in low-grade tumor (G_1,2_) regardless the tumor stage. This statement was based on the observation of insignificant increases in VEGF levels (*P* ≥ 0.2) along with the balanced distributions of VR secretion profiles which were comparable to that of controls (*P* = 0.33). In high-grade tumor (G_3,4_), mostly presented at advanced stage (93.8%), the elevated sVEGFR-1 failed to suppress the VEGF levels as indicated by marked increases in VEGF levels (*P* ≤ 0.002). The distribution of VR secretion profiles was skewed toward V_H_R_H_ and differed significantly from that of controls (*P* = 0.002).

### 3.4. Normality and Abnormality of VEGF/sVEGFR-1 Secretion Patterns

For the purpose of assay characterization, the VEGF/sVEGFR-1 secretion patterns for the entire study population were grouped into eight categories. Firstly, four levels of VEGF concentrations were graded: above 80 percentile rank, 84.5 pg/mL, as very high (V_HH_); between 60 and 80 percentile ranks, 32.5–84.5 pg/mL, as high (V_H_); between 40 and 60 percentile ranks, 23.2–32.5 pg/mL, as intermediate (V_I_); below 40 percentile rank, 23.2 pg/mL, as low (V_L_). Secondly, the associated sVEGFR-1 levels were divided as high (R_H_) and low (R_L_) using the median concentration of 34.5 pg/mL as a cutoff. VEGF/sVEGFR-1 secretion patterns for the healthy subjects and patients with low-grade and high-grade tumors were classified accordingly (Table 5).

 The degree of abnormality/normality for each of the eight VEGF/sVEGFR-1 secretion patterns was rated based on the magnitude of its likelihood ratio (LR) which is a ratio of two proportions: the subset of cancer patients with a particular secretion pattern divided by the subset of normal subjects with the same pattern. A VEGF/sVEGFR-1 secretion pattern was graded as abnormal for a much greater likelihood of being observed in cancer patients than in healthy controls and the reverse was true for a pattern rated as normal. This study in accordance with the standard diagnostic rating scales [31] rated the VEGF/sVEGFR-1 secretion patterns with LRs 4–∞ as definitely abnormal; 2.56 as probably abnormal; 0.8–1.66 as possibly abnormal; 0.3–0.59 as probably normal; 0–0.17 as definitely normal (Table 6). Patterns with intermediate to very high levels of VEGF in association with high sVEGFR-1 level represented abnormal secretion patterns for their high LRs ≥ 4. But patterns with low sVEGFR-1 levels no matter how high the VEGF levels were tended to tilt towards normality with decreasing LRs from 1 to 0. Patients with high-grade tumor had a greater chance (62.5%) to express a definitely abnormal VEGF/sVEGFR-1 secretion pattern than low-grade tumor (20%) and a lower chance (37.6% versus 72%) to be observed with normal secretion pattern (*P* = 0.007). However, without taking levels of sVEGFR-1 into consideration, VEGF alone had much lower diagnostic power in differentiating normal (LRs = 0.3–0.9) from abnormal secretion patterns (LRs = 2.2–2.4).

## 4. Discussion

Using high circulating VEGF levels as an index for disease aggressiveness is based on its association with tumor progression and prognosis [13–20]. However, high false-positive and negative rates were the cause of concern for assay validity [16, 21, 22]. This deficiency might be discerned on how we defined the disease aggressiveness. Advanced clinical stage is generally considered as aggressive disease stage by the TNM criteria. Our study on VEGF regulation by sVEGFR-1 in correlation with stepwise increases in combined tumor grade and stage starting from low grade at an early stage to high grade at the advanced stage revealed that the upregulated sVEGFR-1 in high-grade tumor which mostly presented at advanced stage failed to normalize the VEGF levels leading to persistent VEGF elevation. While the regulative capability of sVEGFR-1 for low-grade tumor in all stages remained effective and was capable of normalizing the VEGF levels in most patients. Based on the observation in our series on HNSCC, combined high grade and advanced stage rather than the advanced stage alone was the significant feature for disease aggressiveness evidenced by VEGF dysregulation.

 Our findings were in line with several studies on experimental tumor models and human tumor specimens. Experimentally, the association of tumor grade and angiogenesis had been demonstrated by a mouse prostate cancer model [32]. Expression of VEGF in tissue and serum was observed in poorly differentiated tumor but not in well- and moderately differentiated tumors. In a human brain tumor model, VEGF has been suggested to be a protagonist in controlling the transition from low-grade glioma to highly vascularized malignant glioma [33]. A high VEGF-to-sVEGFR-1 ratio indicating a VEGF-dominant state was determined for malignant glioblastomas in contrast to the lower ratio, which signified an sVEGFR-1-dominant state, for diffuse astrocytomas [34]. The association of VEGF expression with tumor aggressiveness was also demonstrated in study of a HNSCC cell line transfected with VEGF cDNA [35]. The cell line used for the study was characterized as a low VEGF expressor, having a nonaggressive phenotype for being a well-differentiated and slow growing tumor in vivo. VEGF transfection was sufficient to induce tumor aggressiveness characterized by rapid tumor growth and stromal invasion.

 In assaywise, the VEGF/sVEGFR-1 secretion patterns defined for the entire study population were rated for their degree of normality or abnormality according to their LRs describing the chance of a particular secretion pattern being observed in cancer patients in contrast to healthy controls. Secretion patterns designating high levels of VEGF and sVEGFR-1 were regarded as abnormal for their LRs of ≥4 and were suggestive of VEGF dysregulation as sVEGFR-1 failed to suppress VEGF levels. As expected, more high-grade tumors (62.5%) than the low grade (20%) were observed to express a definitely abnormal VEGF/sVEGFR-1 pattern. Interestingly, patterns with low sVEGFR-1 levels no matter how high the VEGF levels were had low LRs ≤ 1 to signify a normal pattern. Without taking the levels of sVEGFR-1 into consideration, VEGF alone had much lower diagnostic power in differentiating between normal (LRs = 0.3–0.9) and abnormal secretion patterns (LRs = 2.2–2.4). Measurement of sVEGFR-1 could enhance the diagnostic power of VEGF assay particularly in ruling in and ruling out the aggressive disease stage under high VEGF concentrations. High levels of both VEGF/sVEGFR-1 could help identify subset of low-grade tumor with underlying aggressive disease state, while high VEGF levels with low receptor concentrations, which was a normal secretion pattern, observed in a subset of high-grade tumor might indicate the nonaggressive disease state.

 The results from this study suggest that plasma VEGF and sVEGFR-1 could serve as useful biomarkers to help strengthening the prognostic power of the pretreatment circulating VEGF assay used by many investigators [13, 15, 17–20]. Of special interest is the management of tumor hypoxia, an important characteristic of the aggressive malignant phenotype observed in HNSCC [36]. Hypoxia is known to be a condition that activates the expression of VEGF and sVEGFR-1 [23, 30]. The high VEGF/sVEGFR-1 secretion pattern might reflect tumor hypoxia and indicate the justification of incorporating the VEGF targeting agent such as bevacizumab into the chemoradiation regime with the expectation that VEGF inhibition might induce vascular normalization which would lead to improved tumor oxygenation and thereby sensitizing tumor to radiation [37].

## 5. Conclusion

High circulating VEGF levels tend to reflect tumor aggressiveness for being associated with advanced stage and poor prognosis. For HNSCC, high-grade tumor with advanced stage was aggressive compared to low-grade tumor at all stages because of its ineffective VEGF regulation by sVEGFR-1 leading to persistent VEGF elevation. Measurement of sVEGFR-1 helps improve the diagnostic power of the VEGF assay particularly in ruling in and ruling out the aggressive disease state under high VEGF concentrations. VEGF and sVEGFR-1 would serve as useful biomarkers for better prediction of prognosis than VEGF alone and provide information for management of tumor hypoxia.

## Figures and Tables

**Table 1:** Characteristics of the study population.

Characteristic	Patient (*n* = 82)	Normal (*n* = 32)
Age, y		
Mean (range)	58.4 (16–83)	31.7 (22–51)
Sex, *n* (%)		
Male	64 (79)	7 (22)
Female	17 (21)	25 (78)
Primary site, *n* (%)		
Nasopharynx	17 (20.7)	
Oral cavity	23 (28)	
Oropharynx	12 (14.6)	
Hypopharynx	15 (18.3)	
Larynx	15 (18.3)	
T stage, *n* (%)		
T_1-2_	29 (35.4)	
T_3-4_	53 (64.6)	
Node status, *n* (%)		
Negative	40 (48.8)	
Positive	42 (51.2)	
TNM stage, *n* (%)		
I-II	18 (22.)	
III-IV	64 (78)	
Histological grade, *n* (%)		
Well differentiated (G_1_)	31 (37.8)	
Moderately differentiated (G_2_)	19 (23.2)	
Poorly differentiated (G_3_)	22 (26.8)	
Undifferentiated (G_4_)	10 (12.2)	

**Table 2:** Plasma VEGF and sVEGFR-1 levels and VR secretion profile.

Variable	Normal (*n* = 32)	HNSCC (*n* = 82)	*P*
VEGF, pg/mL			
Mean (SD)	41.7 (50.1)	71.1 (133.7)	
Median (IQR)	22.8 (14.5–38.1)	29.4 (19.3–73.9)	0.03
sVEGFR-1, pg/mL			
Mean (SD)	35.3 (30.1)	45.1 (27.7)	
Median (IQR)	27.9 (19.5–34)	37.2 (29.8–54.3)	0.0001
VR secretion profile			0.03
V_H_R_L_, *n* (%)	9 (28.1)	11 (13.6)	
V_H_R_H_, *n* (%)	8 (25)	45 (54.9)	
V_L_R_H_, *n* (%)	10 (31.3)	17 (20.7)	
V_L_R_L_, *n* (%)	5 (15.6)	9 (11)	

**Table 3:** sVEGFR-1 upregulation and VEGF suppression in association with clinicopathological parameters.

Parameter	sVEGFR-1 upregulation, *n* (%)			VEGF suppression, *n* (%)		
	V_H_R_L_	V_H_R_H_	P*	V_H_R_H_	V_L_R_H_	P*
Control	9 (52.9)	8 (47.1)	—	8 (44.4)	10 (55.6)	—
TNM stage						
I, II	3 (33.3)	6 (66.7)	0.43	6 (50)	6 (50)	1
III, IV	8 (17)	39 (83)	0.009	39 (78)	11 (22)	0.02
T stage						
T_1,2_	4 (26.7)	11 (73.3)	0.17	11 (57.9)	8 (42.1)	0.52
T_3,4_	7 (17)	34 (83)	0.009	34 (79)	9 (21)	0.01
Nodal status						
N−	6 (26)	17 (74)	0.29	17 (58.6)	12 (41.4)	0.38
N+	5 (15.2)	28 (84.8)	0.008	28 (84.9)	5 (15.1)	0.003
Tumor grade						
G_1,2_	7 (25.9)	20 (74.1)	0.11	20 (58.8)	14 (41.2)	0.39
G_3,4_	4 (14)	25 (86)	0.007	25 (89.3)	3 (10.7)	0.002

*Comparison between tumor and normal controls.

**Table 4:** VR secretion profile and VEGF and sVEGFR-1 concentrations in correlation with stepwise increase in combined tumor grade and stage.

Tumor grade and stage	VR secretion profile, *n* (%)				VEGF, pg/mL		sVEGFR-1, pg/mL	
	V_H_R_L_	V_H_R_H_	V_L_R_H_	V_L_R_L_	Median (range)	P*	Median (range)	P*
Normal control	9	8	10	5	22.9	—	27.86	—
(*n* = 32)	(28.1)	(25)	(31.3)	(15.6)	(7.1–228.7)		(10.4–147.4)	
G_1,2_ T_1,2_ N−	2	5	6	3	20.6	0.46	32.74	0.04
(*n* = 16)	(12.5)	(31.3)	(37.5)	(18.8)	(10.2–124.6)		(18.1–194)	
G_1,2_ T_3,4_ N−	1	6	5	2	21.6	0.30	43.7	0.004
(*n* = 14)	(7.1)	(42.9)	(35.7)	(14.3)	(1.3–168)		(17.3–171)	
G_1,2_ T_1–4_ N+	4	9	3	4	25.6	0.20	33.32	0.03
(*n* = 20)	(20)	(45)	(15)	(20)	(13.4–1121.9)		(21.2–78)	
G_3,4_ T_2–4_ N−	3	6	1	0	91.5	0.002	35.79	0.04
(*n* = 10)	(30)	(60)	(10)	(0)	(21.3–271.6)		(15.5–54.1)	
G_3,4_ T_3-4_ N+	1	19	2	0	65.4	0.0008	58.84	<.0001
(*n* = 22)	(4.5)	(86.4)	(9.1)	(0)	(17.5–258.5)		(28.8–92.4)	

*Comparison between patients and normal controls.

**Table 5:** VEGF/sVEGFR-1 and VEGF secretion patterns in normal controls and patients with low-grade and high-grade tumors.

VEGF	sVEGFR-1	Normal controls, *n* (%)	Low-grade tumor, *n*	High-grade tumor, *n* (%)
pg/mL	pg/mL	(*n* = 32)	(%) (*n* = 50)	(*n* = 32)
Very high, V_HH_ > 84.5	High, R_H_ ≥ 34.5	0 (0)	3 (6)	7 (21.9)
	Low, R_L_ < 34.5	5 (15.6)	3 (6)	5 (15.6)

	Total	5 (15.6)	6 (12)	12 (37.5)

High, V_H_ 32.5–84.5	High, R_H_ ≥ 34.5	1 (3.1)	4 (8)	9 (28.1)
	Low, R_L_ < 34.5	4 (12.5)	3 (6)	2 (6.3)

	Total	5 (15.6)	7 (14)	11 (34.4)

Intermediate, V_I_ 23.2–32.5	High, R_H_ ≥ 34.5	1 (3.1)	7 (14)	4 (12.5)
	Low, R_L_ < 34.5	4 (12.5)	6 (12)	0 (0)

	Total	5 (15.6)	13 (26)	4 (12.5)

Low, V_L_ < 23.2	High, R_H_ ≥ 34.5	5 (15.6)	13 (26)	3 (9.4)
	Low, R_L_ < 34.5	12 (37.5)	11 (22)	2 (6.3)

	Total	17 (53.1)	24 (48)	5 (15.6)

**Table 6:** Likelihood ratios (LR), degree of normality/abnormality for VEGF/sVEGFR-1 (V/R), and VEGF (V) secretion patterns.

Test result	Low-grade tumor (*n* = 50)				High-grade tumor (*n* = 32)			
	V/R, *n* (%)	LR	V, *n* (%)	LR	V/R, *n* (%)	LR	V, *n* (%)	LR
Definitely	V_HH_R_H_, 3 (6)	∞	—	—	V_HH_R_H_, 7 (21.9)	∞	—	—
abnormal	V_I_R_H_, 7 (14)	4.47			V_H_R_H_, 9 (28.1)	9		
					V_I_R_H_, 4 (12.5)	4		
Probably	V_H_R_H_, 4 (8)	2.56	—	—	—	—	V_HH_, 12 (37.5)	2.42
abnormal							V_H_, 11 (34.4)	2.2
Possibly	V_L_R_H_, 13 (26)	1.66	V_HH_, 6 (12)	0.8	V_HH_R_L_, 5 (15.6)	1	V_I_, 4 (12.5)	0.8
abnormal	V_I_R_L_, 6 (12)	0.96	V_H_, 7 (14)	0.9				
			V_I_, 13 (26)	1.66				
			V_L_, 24 (48)	0.9				
Probably	V_HH_R_L_, 3 (6)	0.38	—	—	V_H_R_L_, 2 (6.3)	0.5	V_L_, 5 (15.6)	0.3
normal	V_H_R_L_, 3 (6)	0.48			V_L_R_H_, 3 (9.4)	0.6		
	V_L_R_L_, 11 (22)	0.59						
Definitely	—	—	—	—	V_I_R_L_, 0 (0)	0	—	—
normal					V_L_R_L_, 2 (6.3)	0.17		

## References

[1] Jemal A, Siegel R, Ward E, Murray T, Xu J, Thun MJ (2007). Cancer statistics, 2007. *CA: A Cancer Journal for Clinicians*.

[2] de Bree R, Deurloo EE, Snow GB, Leemans CR (2000). Screening for distant metastases in patients with head and neck cancer. *Laryngoscope*.

[3] Kowalski LP, Medina JE (1998). Nodal metastases: predictive factors. *Otolaryngologic Clinics of North America*.

[4] Salesiotis AN, Cullen KJ (2000). Molecular markers predictive of response and prognosis in the patient with advanced squamous cell carcinoma of the head and neck: evolution of a model beyond TNM staging. *Current Opinion in Oncology*.

[5] Folkman J (2002). Role of angiogenesis in tumor growth and metastasis. *Seminars in Oncology*.

[6] Kyzas PA, Cunha IW, Ioannidis JPA (2005). Prognostic significance of vascular endothelial growth factor immunohistochemical expression in head and neck squamous cell carcinoma: a meta-analysis. *Clinical Cancer Research*.

[7] Seiwert TY, Cohen EEW (2008). Targeting angiogenesis in head and neck cancer. *Seminars in Oncology*.

[8] Mǎrgǎritescu C, Pirici D, Simionescu C (2009). VEGF and VEGFRs expression in oral squamous cell carcinoma. *Romanian Journal of Morphology and Embryology*.

[9] Sauter ER, Nesbit M, Watson JC, Klein-Szanto A, Litwin S, Herlyn M (1999). Vascular endothelial growth factor is a marker of tumor invasion and metastasis in squamous cell carcinomas of the head and neck. *Clinical Cancer Research*.

[10] O-charoenrat P, Rhys-Evans P, Eccles SA (2001). Expression of vascular endothelial growth factor family members in head and neck squamous cell carcinoma correlates with lymph node metastasis. *Cancer*.

[11] Boonkitticharoen V, Kulapaditharom B, Leopairut J (2008). Vascular endothelial growth factor A and proliferation marker in prediction of lymph node metastasis in oral and pharyngeal squamous cell carcinoma. *Archives of Otolaryngology*.

[12] Grunewald M, Avraham I, Dor Y (2006). VEGF-induced adult neovascularization: recruitment, retention, and role of accessory cells. *Cell*.

[13] Bass MB, Sherman SI, Schlumberger MJ (2010). Biomarkers as predictors of response to treatment with motesanib in patients with progressive advanced thyroid cancer. *Journal of Clinical Endocrinology and Metabolism*.

[14] Shang ZJ, Li JR, Li ZB (2007). Upregulation of serum and tissue vascular endothelial growth factor correlates with angiogenesis and prognosis of oral squamous cell carcinoma. *Journal of Oral and Maxillofacial Surgery*.

[15] Teknos TN, Cox C, Yoo S (2002). Elevated serum vascular endothelial growth factor and decreased survival in advanced laryngeal carcinoma. *Head and Neck*.

[16] Qian CN, Zhang CQ, Guo X (2000). Elevation of serum vascular endothelial growth factor in male patients with metastatic nasopharyngeal carcinoma. *Cancer*.

[17] Bernaards C, Hegde P, Chen D (2010). Circulating vascular endothelial growth factor (VEGF) as a biomarker for bevacizumab-based therapy in metastatic colorectal, non-small cell lung, and renal cell cancers: analysis of phase III studies. *Journal of Clinical Oncology*.

[18] Hanrahan EO, Ryan AJ, Mann H (2009). Baseline vascular endothelial growth factor concentration as a potential predictive marker of benefit from vandetanib in non-small cell lung cancer. *Clinical Cancer Research*.

[19] Poon RTP, Lau C, Pang R, Ng KK, Yuen J, Fan ST (2007). High serum vascular endothelial growth factor levels predict poor prognosis after radiofrequency ablation of hepatocellular carcinoma: importance of tumor biomarker in ablative therapies. *Annals of Surgical Oncology*.

[20] Hefler LA, Zeillinger R, Grimm C (2006). Preoperative serum vascular endothelial growth factor as a prognostic parameter in ovarian cancer. *Gynecologic Oncology*.

[21] Kuemmel S, Thomas A, Landt S (2009). Circulating vascular endothelial growth factors and their soluble receptors in pre-invasive, invasive and recurrent cervical cancer. *Anticancer Research*.

[22] Duque JLF, Loughlin KR, Adam RM, Kantoff P, Mazzucchi E, Freeman MR (2006). Measurement of plasma levels of vascular endothelial growth factor in prostate cancer patients: relationship with clinical stage, gleason score, prostate volume, and serum prostate-specific antigen. *Clinics*.

[23] Shibuya M (2006). Differential roles of vascular endothelial growth factor receptor-1 and receptor-2 in angiogenesis. *Journal of Biochemistry and Molecular Biology*.

[24] Bailey AP, Shparago M, Gu JW (2006). Exercise increases soluble vascular endothelial growth factor receptor-1 (sFlt-1) in circulation of healthy volunteers. *Medical Science Monitor*.

[25] Toi M, Bando H, Ogawa T, Muta M, Hornig C, Weich HA (2002). Significance of vascular endothelial growth factor (VEGF)/soluble VEGF receptor-1 relationship in breast cancer. *International Journal of Cancer*.

[26] Yamaguchi T, Bando H, Mori T (2007). Overexpression of soluble vascular endothelial growth factor receptor 1 in colorectal cancer: association with progression and prognosis. *Cancer Science*.

[27] Chang YT, Chang MC, Wei SC (2008). Serum vascular endothelial growth factor/soluble vascular endothelial growth factor receptor 1 ratio is an independent prognostic marker in pancreatic cancer. *Pancreas*.

[28] Kendall RL, Thomas KA (1993). Inhibition of vascular endothelial cell growth factor activity by an endogenously encoded soluble receptor. *Proceedings of the National Academy of Sciences of the United States of America*.

[29] Maynard SE, Min JY, Merchan J (2003). Excess placental soluble fms-like tyrosine kinase 1 (sFlt1) may contribute to endothelial dysfunction hypertension, and proteinuria in preeclampsia. *Journal of Clinical Investigation*.

[30] Barleon B, Siemeister G, Martiny-Baron G, Weindel K, Herzog C, Marmé D (1997). Vascular endothelial factor up-regulates its receptor fms-like tyrosine kinase 1 (FLT-1) and a soluble variant of FLT-1 in human vascular endothelial cells. *Cancer Research*.

[31] Sackett DL, Haynes RB, Guyatt GH, Tugwell P (1991). *Clinical Epidemiology: A Basic Science for Clinical Medicine*.

[32] Huss WJ, Hanrahan CF, Barrios RJ, Simons JW, Greenberg NM (2001). Angiogenesis and prostate cancer: identification of a molecular progression switch. *Cancer Research*.

[33] Plate KH, Risau W (1995). Angiogenesis in malignant gliomas. *Glia*.

[34] Lamszus K, Ulbricht U, Matschke J, Brockmann MA, Fillbrandt R, Westphal M (2003). Levels of soluble vascular endothelial growth factor (VEGF) receptor 1 in astrocytic tumors and its relation to malignancy, vascularity, and VEGF-A. *Clinical Cancer Research*.

[35] Detmar M, Velasco P, Richard L (2000). Expression of vascular endothelial growth factor induces an invasive phenotype in human squamous cell carcinomas. *American Journal of Pathology*.

[36] Koukourakis MI, Giatromanolaki A, Sivridis E (2002). Hypoxia-inducible factor (HIF1A and HIF2A), angiogenesis, and chemoradiotherapy outcome of squamous cell head-and-neck cancer. *International Journal of Radiation Oncology Biology Physics*.

[37] Riesterer O, Milas L, Ang KK (2008). Combining molecular therapeutics with radiotherapy for head and neck cancer. *Journal of Surgical Oncology*.

